# Drop splitting on hydrophobic wedge-shaped tips after central impact: effect of sharpness and wetting properties†

**DOI:** 10.1039/d4sm01373e

**Published:** 2025-02-07

**Authors:** Xiaoteng Zhou, Diego Diaz, Zhongyuan Ni, Sajjad Shumaly, Jie Liu, Michael Kappl, Hans-Jürgen Butt

**Affiliations:** a Max Planck Institute for Polymer Research Ackermannweg 10 55128 Mainz Germany kappl@mpip-mainz.mpg.de butt@mpip-mainz.mpg.de; b KTH Royal Institute of Technology SE-100 44 Stockholm Sweden; c Beijing National Laboratory for Molecular Science, Key Laboratory of Green Printing, Institute of Chemistry, Chinese Academy of Sciences Beijing 100190 P. R. China; d School of Chemical Sciences, University of Chinese Academy of Sciences Beijing 100049 P. R. China

## Abstract

Drop impact on a wedged structure is a common phenomenon in daily life and industry. Although drop impact has been studied extensively since high-speed cameras have become available, little is known about drop impact on wedge tips of these structures. Here, we combine experiments and volume-of-fluid simulations to determine how velocity, the sharpness of the structure, and the surface wettability influence the outcome. The central impact of water drops onto wedge tips coated with superhydrophobic nanofilaments or with hydrophobic polystyrene (PS) was imaged. On superhydrophobic surfaces, drops fully rebound or split after impact. On hydrophobic PS surfaces, drops are deposited or split. A critical Weber number (We) was used to describe the transition between deposition/rebounding and splitting. It increases with the top width of the wedge tip and its top angle. The critical We and drop behavior is also affected by wetting properties which determine the drop adhesion and lateral drop friction. Our investigations may help to design new structures to prevent icing or produce tiny drops efficiently in applications.

## Abstract 2

Does a knife-like wedge-shaped tips always split a centrally impacting water drop?  Here, we combine experiments and volume of fluid (VOF) simulations to investigate this process. The answer might surprise you—find out in this manuscript.

## Graphical abstract

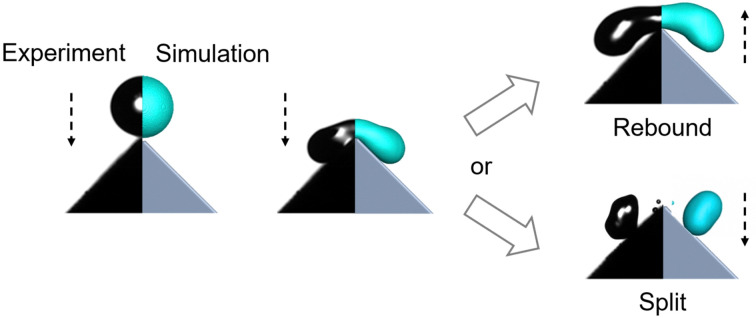

## Introduction

Drop impact is an important interfacial phenomenon, present in nature and in various industrial applications. When drops hit a surface they can be deposited,^1^ rebound^2,3^ or splash.^4,5^ The outcome of drop impact is determined by the liquid and surface properties.^6^ For liquids, these properties are mainly the surface tension, viscosity, drop size and kinetic impact energy. Surface structure and wettability are also crucial for the resulting drop impact dynamics. Drop impact is important since it determines the degree of exchange in momentum, mass, and energy between a drop and a solid surface.^2,3,7^ Researchers have established a universal principle for describing the drop contact behavior and have been working on various ways to realize fast drop detachment for applications in industry.^3,7–11^ However, drop splitting, another fundamental question of the drop impact phenomenon, has received less attention. Splitting occurs when a drop breaks up into two or more smaller drops after impacting a protuberant sharp structure or the sharp wedge tip of a surface.^12^ This process is a common phenomenon in nature,^13^ where surfaces generally are far from being flat, with ridges that favor the occurrence of instabilities and affect drop dynamics.

Drop splitting occurs on flat surfaces or surfaces with microstructures at a high kinetic energy.^14–19^ When the surface is flat, a drop splits into ejecting satellite drops, usually denoted as drop splash.^20,21^ If the impact velocity is sufficiently high, such splashing occurs at the wedge tip of a spreading lamella, which leads to splitting in the radial direction.^22^ It was demonstrated that at sufficiently high kinetic energy, water drops split into two when hitting a superhydrophobic wire-like ridge on a horizontal surface.^23^ Superhydrophobic wires have also been used to cut falling water drops in half.^24^ These superhydrophobic cylinders had radii of curvature of 0.1–0.25 mm. Liu *et al.* analyzed the impact of water drops on *Echevaria* leaves.^25^ These leaves have curved surfaces with typical radii of curvatures (few mm) leading to asymmetric bouncing of impacting water drops and a reduction of the contact time at high We. At low We, Tang *et al.* found drops rebounding from cones with superhydrophobic coatings,^26^ and Ramirez-Soto *et al.* observed water drops rebounding from oil drops on superamphiphobic surfaces after central impact.^27^ Using simulations of rebounding drops, Yoon *et al.* found that a substantial part of the energy of rebounding drops at low We is dissipated in capillary waves.^28^ A clear understanding of when drops split by shaped tips after central impact with varied wettability is still missing. More specifically, a universal description of the splitting process in terms of its main controlling physical parameters is needed. Such knowledge helps in the improvement of a variety of applications. For example, drop splitting can increase the area of the gas–liquid interface of a system, so the efficiency of heat and mass transfer can be improved.^29,30^ It could also be beneficial for improved inkjet printing,^31,32^ microfluidic performance,^33,34^ energy harvesting,^35^ and understanding the icing process after a drop is separated into pieces on the surface of power transmission equipment^36^ or aircraft.^37^

**Fig. 1:**
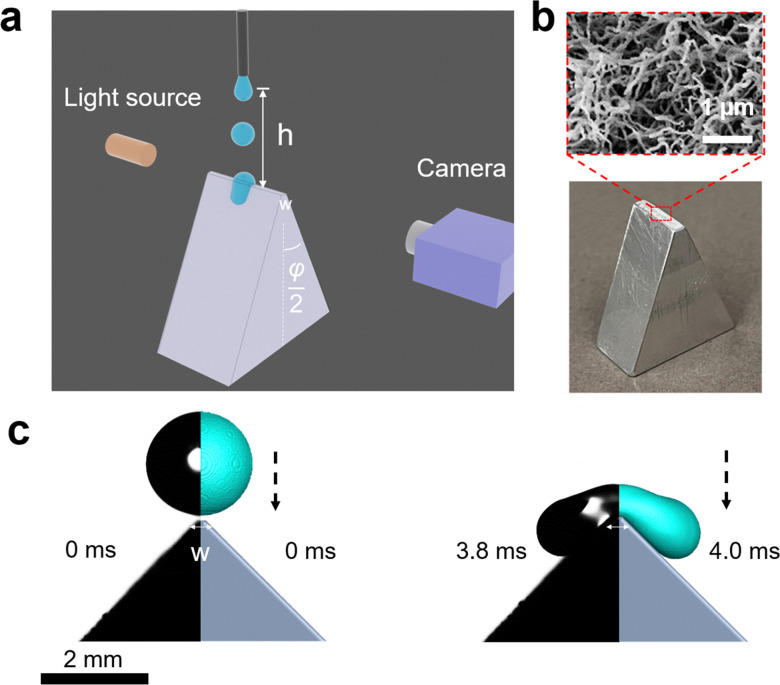
Recording of the drop splitting process. (a) Scheme to show the experimental setup and the parameters. (b) Optical picture showing the wedge-like structure we used with *w* = 2.0 mm and *φ* = 90°. The scanning electron micrograph in the red frame shows the nanofilament coating for superhydrophobic cases. (c) Two snapshots of drop motion during the impact on a superhydrophobic wedge with top angle *φ* = 90° as an example before rebounding or splitting with We is 5. The drop movement is characterized by both experiments (left, black/grayscale) and simulation (right, grey/cyan).

Here, we systematically study the splitting of drops impacting on sharp wedge tips. The influence of the impact speed, wedge tip sharpness, and wetting properties of the surfaces was investigated. Our results reveal that the splitting threshold is determined by both the geometry of the wedge tip surface and the wetting property of the surface coating (Fig. 1). The experimental data are complemented by numerical simulations based on the volume of fluid (VOF) method to determine an exact boundary condition for a split (ESI,† Section S1).^38,39^

## Experimental section

Drops of deionized water (18 MΩ cm resistivity) with a radius of 1 mm (drop volume is around 4.1 μL) were released from heights between 1 to 9 cm onto wedge tip surfaces coated with superhydrophobic silicone nanofilaments (NF) or polystyrene (PS). The temperature was 20 ± 3 °C and the air humidity was from 30% to 40%. To dispense drops, a motorized syringe pump was used. We recorded the process with a high-speed camera (Photron UX100, 5000 fps, Fig. 1). The impact velocity when the drop contacted the surface was calculated by 
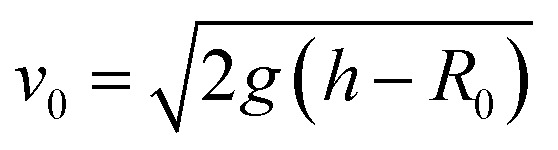
, where *h* is the release height. We define this height as the distance between the initial center of the drop and the tip of the wedge tip surface. This calculated impact velocity agrees well with the velocity in the recording videos obtained from two nearby frames when the drop contacts the surface.

We machined prismatic aluminum surfaces with a flat horizontal top of width *w* (Fig. 1). The vertical height of the shaped structure from the top wedge tip to the bottom was more than 1 cm. The structures were defined by the combination of top angle *φ*, and the top width *w*. Structures with top angles *φ* = 10°, 45° and 90° and top widths of *w* = 0.2 ± 0.03 mm and 2.0 ± 0.02 mm were fabricated. In addition, we used vertical thin glass sheets (Menzel Gläser, VWR company, *φ* = 0°), where the top width was given by the sheet thickness of 0.18 ± 0.02 mm. All substrates were cleaned with ethanol to remove organic residues.

Superhydrophobic surfaces were prepared using silicone nanofilament coating. First, the surface was activated by an oxygen plasma (Femto low-pressure plasma system, Diener electronic, 100 W) for 10 min. 400 μL trichloromethylsilane was dissolved in toluene (with 200 ppm water content).^40^ The plasma-treated surfaces were then immersed in this solution for 12 h, which leads to the spontaneous growth of silicone nanofilaments on the surfaces. Coated surfaces were washed with toluene 3 times and dried by nitrogen gas. The static contact angle of 6 μL water was 158° ± 2° with a contact angle hysteresis lower than 10°. Hydrophobic surfaces were prepared by dip-coating the wedge tip structures into a solution consisting of 20% (weight) of polystyrene (molecular weight, 192 kg mol^−1^, Sigma-Aldrich) in toluene. After moving the structures at a speed of 90 mm min^−1^ into the solution and waiting for 10 s, the substrates were moved up again at a speed of 10 mm min^−1^. Finally, the films were annealed in an oven at 120 °C under a vacuum for 24 hours. 6 μL water drops had an advancing contact angle of 93° ± 2° and receding contact angle of 82° ± 1° on a glass substrate and advancing contact angle of 100° ± 2° and receding contact angle of 86° ± 2°.

To measure the dynamic contact angle and its velocity as a drop splits into two parts and slides on a surface, we refined the 4S-SROF method.^41^ The script we developed is available on GitHub for use.^42^

## Volume of fluid (VOF) simulations

Three-dimensional numerical simulations of drops impacting on a wedge tip structure were conducted using the volume of fluid method on the open-source CFD platform OpenFoam (Fig. S1, ESI†). The governing equations in the numerical simulations were continuity, momentum and phase fraction equations.^38,39^

1 ∇·*v⃑* = 0

2
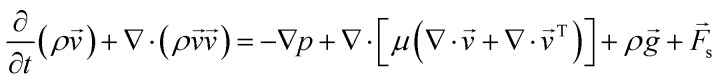
3
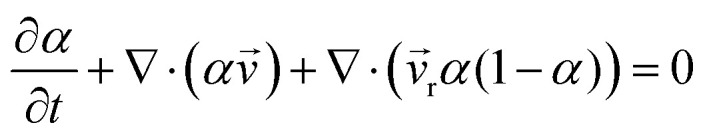
Here, *v⃑* is the velocity vector, *t* is time, *p* is pressure and *g⃑* is the acceleration of gravity. The phase fraction *α* is defined as the volume fraction of the liquid phase in each grid. The fluid density *ρ* and viscosity *μ* were calculated as,

4 *ρ* = *αρ*_water_ + (1 − *α*)*ρ*_air_

5 *μ* = *αμ*_water_ + (1 − *α*)*μ*_air_

The surface tension force *F⃑*_s_ can be determined by the continuum surface force (CSF) model:

6 *F⃑*_s_ = *σκ*∇*α*

where *σ* is the surface tension, and *κ* is the interface curvature. *κ* is given as:7
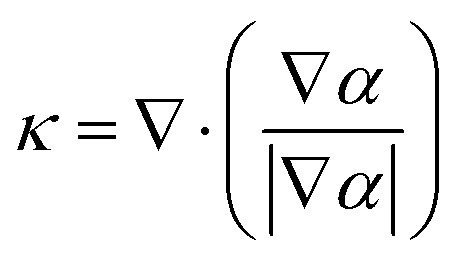


To sharpen the interface, the extra compression term 
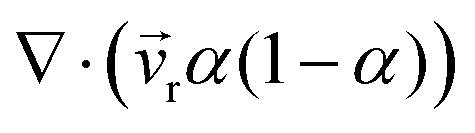
 is introduced in the phase fraction equation (*i.e.*, eqn (3)), where *U⃑*_r_ is the artificial compression velocity. The PISO method (pressure implicit with splitting of operators) is used for the pressure velocity coupling. The residual errors of the pressure, phase fraction and velocity are all set as 10^−8^. The adaptive time steps in the simulation are adjusted by the maximum Courant number (Co = max(|*v*|δ*t*/δ*x*), where δ*t* and δ*x* are the time step and mesh size, respectively), where the maximum Courant number is set as 0.2.

Fig. S1(a) (ESI†) shows the schematic of the drop impacting on the wedge tip structure. Fig. S1(b) (ESI†) shows the computational domain with size of 6 × 4 × 8 mm^3^. The wedge tip structure surface and bottom surface are set as a no-slip wall with a static contact angle of 180°, the left surface is set as the symmetry boundary, and another surface is set as the pressure outlet boundary. At the beginning of the simulation, a drop with a diameter of 2 mm is placed directly above the wedge tip structure at a certain velocity. Considering the computational efficiency, the grid number is chosen as 3.3 million, where the minimum grid length is about 10 μm. In all simulations, the boundary condition was set to no-slip at the solid–liquid interface. Even for superhydrophobic surfaces we neglected slip, considering that the effective slip is lower than the typical spacing between surface structures.^43^ The nanofilaments had a spacing lower than 0.5 μm. For the superhydrophobic surfaces we neglected the nanoscale roughness and assumed a contact angle of 180° at the grids near the wedge tips.

## Results and discussion

**Fig. 2:**
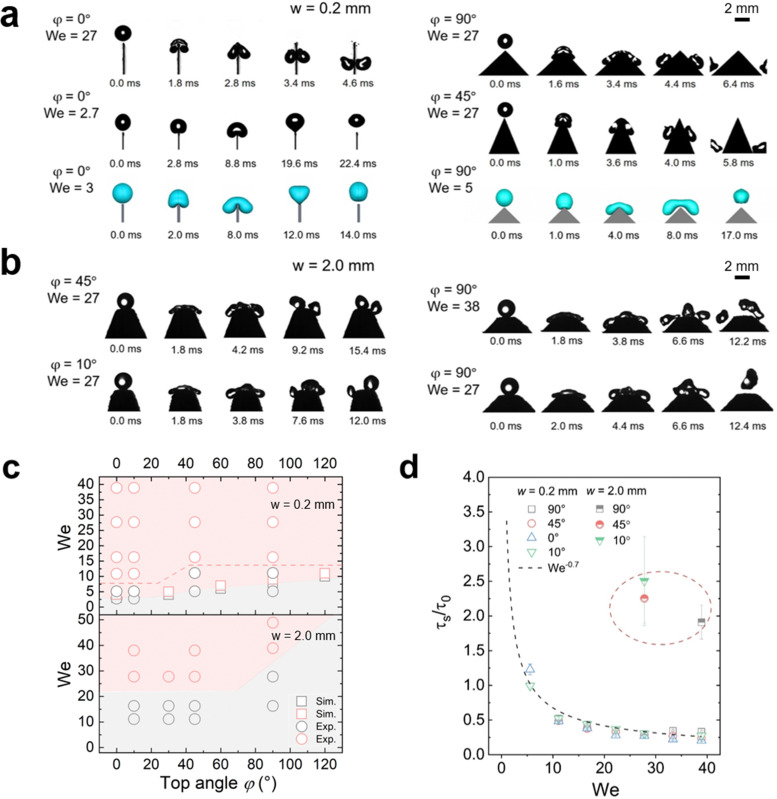
Drop splitting on the nanofilament-coated superhydrophobic surfaces. Image series of water drops (radius = 1 mm) impacting and splitting on nanofilament-coated superhydrophobic surfaces with top width 0.2 mm (a) and 2.0 mm (b) from both experiments and simulations. (c) Diagram showing how We and the top angle of the wedge structure determine drop splitting with different top widths. The red area indicates drop splitting, and the grey areas are for rebounding drops. The red dash line indicates the limit based on experimental data. (d) Splitting time *versus* Weber number We for superhydrophobic surfaces with different top angles *φ* and top widths *w*.

When a water drop impacted on the tip of a sharp-wedged superhydrophobic surface (*w* = 0.2 mm), two scenarios were observed: bouncing and splitting (Fig. 2(a) and Movie S1, ESI†), depending on the Weber number We = *ρR*_0_*v*_0_^2^/*γ* = 2*ρR*_0_*gh*/*γ*. Here, *ρ* is the water density, *γ* is the surface tension, *g* = 9.81 m s^−2^ is the acceleration of free fall, *R*_0_ = (3*V*/4π)^1/3^ is the initial drop radius, and *V* is the drop volume. For drop impact, the Weber number is commonly defined to express the ratio of kinetic energy to surface energy of the drop.

Even for a top width *w* of only 0.2 mm and a top angle *φ* of 0°, bouncing still occurred on superhydrophobic surfaces at We <5 (Fig. 2(a)). Bouncing could be reproduced by VOF simulations assuming a contact angle of 180° and ignoring the surface roughness (Fig. 2(a)). When increasing We to ≈10 or higher, splitting took place (Fig. 2(a) and (c)). When the top width increased to 2 mm, a higher We was required to cause splitting (Fig. 2(b), (c) and Fig. S2, ESI†). Once the contact line spreads beyond the wedge tips, the liquid starts to move downwards. The drops tended to split on both sides of the top area (Fig. 2(b) and Movie S2, ESI†). But if We is not high enough, the wedge part only results in more drop deformation. In an ideal case with quite high We, *e.g.* 38, a middle drop and two drops at each side may appear after splitting, but the middle drop always prefers to merge with one of the side drops after rebounding due to the short distance between them (Fig. 2(b)). Thus, when observing the outcome of such a drop impact, the two resulting drops were quite different in mass and their momentum was not symmetric.

The outcome of drop impact experiments can be plotted in a diagram of We and top angle *φ* (Fig. 2(c)). We see that there is a boundary difference for the splitting cases on the surfaces with different top widths. On 2.0 mm samples with the same top angle *φ*, a drop needs more kinetic energy to split. And with the same *w* value, a higher top angle usually requires a higher kinetic energy to induce splitting. When *w* = 0.2 mm, drops started to split once We > 5 at *φ* = 0°. This critical value of We increased with the top angle value.

For higher top angles, the experimental critical We was usually higher than the simulation result. For example, at *φ* = 90°, the critical We from the experiments was around 16 and in simulations it was 7. Experimental contact widths in front and side view before the splitting (Fig. S3 and S4, ESI†) agree well with the simulation during contact. Only close to the detachment, experiments show a larger contact width than simulation. A possible reason for this discrepancy is the idealized boundary condition in the simulations. For the simulations we used a no-slip boundary condition and a contact angle of 180°. In the experiments, the detachment is not ideal because the liquid penetrates into the nanostructure of the superhydrophobic coating. When comparing the impact of drops on superhydrophobic surfaces with ideal simulation and a real rough surface, the outcome may show different types of drop impacts even though the apparent contact angles are similar,^44^ because the specific nano- and microstructure of the surface also plays an important role. As a result, more kinetic energy is required to trigger splitting in experiments. Such a penetration also led to a difference between simulation and experiments for 2 mm top width. Thus, we do not show the simulation related to the critical We of 2 mm cases here.

To better understand the effect of wedge tip structure on the dynamic process we analyzed the splitting time. It was defined by the time between the first contact of the drop with the wedge tip and the breaking of the liquid bridge between the two separate drops, *τ*_s_. A short splitting time indicates that the drops rapidly detach from the surface. The splitting time decreased with increasing We and scaled as *τ*_s_ ∝ *τ*_0_We^−0.7^ for all surfaces (Fig. 2(d)). Here, we scaled the splitting time by *τ*_0_ = (*ρD*_0_^3^/*γ*)^1/2^. *τ*_0_ is the characteristic contact time of a drop bouncing from a flat surface^2^, with *D*_0_ = 2*R*_0_. The splitting time did not depend on the top angle (Fig. 2(d)). Moreover, the splitting time for *w* = 2.0 mm (∼2*τ*_0_) was roughly two times higher than the contact time of the same drop on a flat nanofilament-coated surface (Fig. 2(d)); for a flat surface we measured 10.6 ms and calculated 9.5 ms. In contrast, on the 0.2 mm samples the contact time was two times faster than on flat surfaces. Moreover, when simulating drop impact for *φ* = 0°, the minimal Weber number for drop splitting also depends on the top width. When increasing *w* from 0.2 to 0.6 mm, the critical We for drop splitting increased from 4 to 8 (Fig. S2, ESI†). Thus, the top width *w* has a stronger influence on drop splitting than the top angle.

The influence of top widths and top angles for superhydrophobic surfaces can be understood by analyzing the energy change. A prerequisite to produce drop splitting into two smaller drops is an initial impact energy 
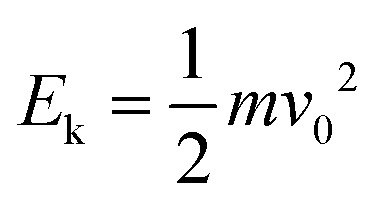
 that surpasses the change in surface energy by splitting and the dissipation *E*_dis_ during this process. Assuming an initial spherical shape of the drops, the change in surface energy between initial (single drop) and final state (two drops at constant volume) is at least Δ*E*_s_ = 4π*R*_0_^2^*γ*(2^1/3^ − 1). The surface energy changes by a factor 2^1/3^ = 1.26. Drops can only split when *E*_k_ > Δ*E*_s_ + *E*_dis_, which determines a lower critical We for splitting.

**Fig. 3:**
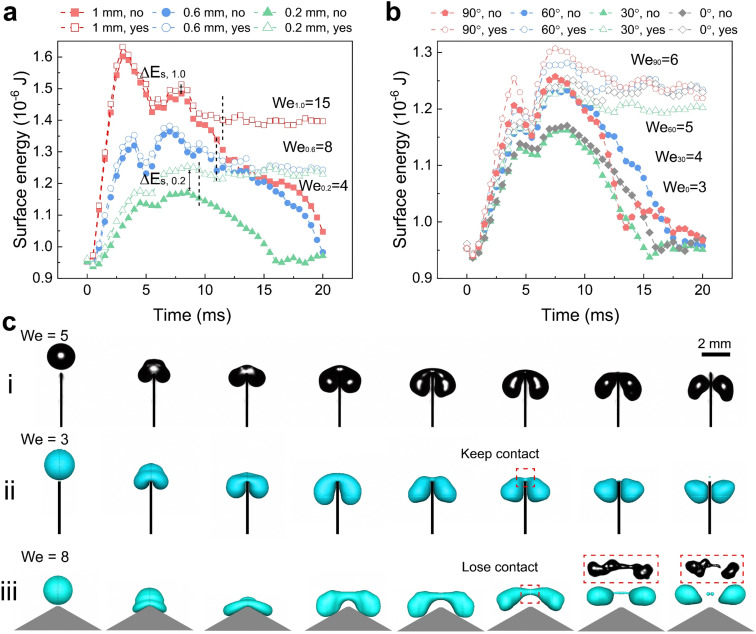
Surface energy *versus* time of the water drops from simulation. (a) Surface energy on surfaces with a top angle *φ* of 0° but different top widths *w versus* time after contact. Vertical dashed lines indicated the time when the drop starts to split. (b) Surface energy on surfaces with a top width *w* of 0.2 mm but different top angles *φ versus* time. For the splitting cases, the surface energy is a sum of two or three drops. (c) The image series of a drop with radius of 1 mm impact process on the superhydrophobic structure with *w* of 0.2 mm and *φ* of 0° (i) and (ii), and *w* of 0.2 mm and *φ* of 120° (iii). The red frame in (iii) is captured from a splitting after rebounding when the drop impacts the side of an ideal non-sharp cylinder shape for comparison. The We shown here expressed the critical We for splitting.

Using simulations, we could trace the exact change in total surface energy during drop splitting or bouncing by summing the energy in each grid covering a drop surface. The simulations shown in Fig. 3(a) were carried out around the critical Weber number for drop splitting. One was done at a slightly higher Weber number (open symbols) so that the drops split. The other was carried out at a slightly lower Weber number (filled symbols) so that the drops bounced. The difference in surface energy changes case by case related to how the spreading proceeded and how the liquid bridge broke up resulting in a splitting. Splitting and bouncing involved intermediate states that had a higher surface area than the initial value (Fig. 3(a) and (b)). In the case of splitting both small drops generated were far from being spherical. During the process, energy is dissipated by internal viscous flow. For a drop with a radius of 1 mm, the initial surface energy is 0.93 μJ. For a top angle *φ* = 0°, and the surface energy in the first 4 ms increased until a maximum was reached (Fig. 3(a)). This maximal energy depends on the top width. For a top width of 1.0 mm (red symbols Fig. 3(a)), the maximum surface energy is a factor of 1.7 times the initial surface energy of 0.93 μJ. For 0.6 mm and 0.2 mm (blue and green symbols Fig. 3(a)), it decreased to less than 1.3. A higher factor of 1.7 for width for the 1.0 mm top width is closer to a splitting into three drops because the much wider top area prefers to cut the drop in the positions of two corners. This tendency is also observed in experiments of the case with *w* = 2.0 mm (Movie S2, ESI†). In the other two cases, it is splitting into two drops. Here, Δ*E*_s_ for splitting in three drops of equal size is 4π*R*_0_^2^*γ*(3^1/3^ − 1) with a factor of around 1.45, which agrees with the simulation. For a top width of 0.2 mm, kinetic energy almost equal to Δ*E*_s_ was required, but no extra energy was needed. In contrast, for *w* > 0.2 mm a threshold energy, like an activation barrier, had to be overcome. When increasing the top angle to *φ* ≥ 60° an energy barrier developed again (Fig. 3(b)).

**Fig. 4:**
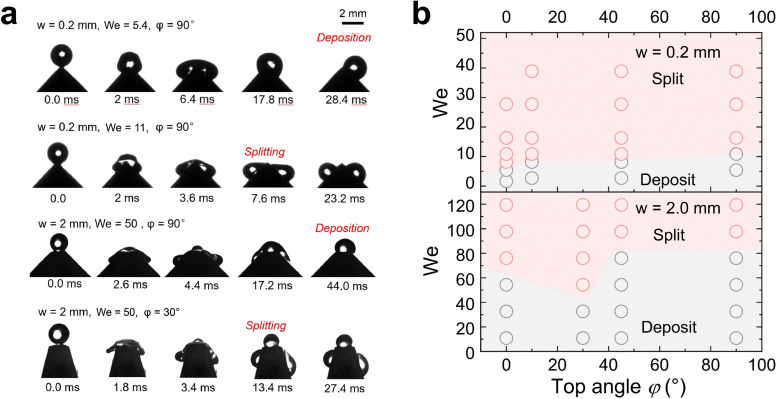
Drop splitting on polystyrene-coated hydrophobic surfaces. (a) Representative image series of water drop (radius = 1 mm) impacting and splitting on PS surfaces with top widths of 0.2 mm and 2.0 mm. (b) Diagram to show how We and the top angle of the wedge tip structure determine drop splitting or deposition on hydrophobic surfaces. The red area indicates drop splitting, and the grey areas are for deposited drops.

Based on the simulations and high-speed videos, two types of splitting processes on superhydrophobic sharp wedge tips can be distinguished. In the first case (Fig. 3(c)-i, ii and Movie S3, ESI†), the liquid always contacts the solid part (red frame in Fig. 3(c)-ii) while the sharp wedge tip penetrates the bridge between the two parts. In the other case, the drop rebounds before the two parts have separated. Splitting happens after the drop has lost contact with the surface (Fig. 3(c)-iii and Fig. 4(b), Movie S3, ESI†). The first process dominates for sharp wedge tips (*φ* ≤ 30°, *w* = 0.2 mm). The second dominates cases with higher top angles (*φ* ≥ 60°) or wider top width (*w* ≥ 0.6 mm) in an ideal case in which liquid does not penetrate the nanofilament structure.

In contrast to superhydrophobic wedge tips, drops do not rebound from hydrophobic PS coated wedge tips. Here, we coated the surface with 1 μm thick PS film using a 20% solution in toluene by dip-coating. Because of the low viscosity of the solution and the slow pull-off process, such a coating does not result in non-uniformity of the wedge tip part significantly as that for the nano-scale films.^45^ Either the complete drop or the two drops produced after splitting were deposited on the surface (Fig. 4 and Movie S4, ESI†). Drops deposit because of the lower contact angle, which leads to a higher adhesion and larger contact area. For *w* = 2.0 mm, again splitting into three drops could be observed. On hydrophobic surfaces, drop splitting took up to 40% longer than on superhydrophobic ones (Fig. 2(d)). Furthermore, the contact line velocity determined from high speed videos^41^ was lower (Fig. S5 and S6, ESI†) on hydrophobic surfaces, especially for the non-splitting case (Fig. S7, ESI†).

The contact angles during drop impact for different wetting properties can be analyzed by side-view videos of the water drops by a resolution-enhancing method (ESI,† Section S2 and Fig. S8).^41^ Lower contact angle change for hydrophobic cases illustrating that the dissipation inside the drop is lower than that in superhydrophobic cases, resulting in a lower critical We for splitting when *w* = 0.2 mm. In all non-split cases, the contact angle usually changes more obviously illustrating more energy dissipation from contact line friction and drop deformation. Therefore, it reduces the probability of the drop breaking apart after rebounding or depositing. But the velocity dependence of contact angles for this kind of impact is far away from previous theories^46^ because the drop is confined by the structure in the direction of gravity. Thus, we don’t discuss the details of dynamic contact angle change here.

## Conclusion

In this work, we explored the splitting of water drops impacting onto superhydrophobic and hydrophobic wedge tips with different top widths *w* and top angles *φ* by both experiments and simulations. At low We, drops rebound from superhydrophobic surfaces and are deposited on hydrophobic surfaces. At high We, drops split. The critical Weber number where bouncing (or deposition) changes to splitting increases with the top width and the top angle. For superhydrophobic surfaces, drop splitting can occur while the drop is still in contact with the wedge tip (for low *φ* value) or after the drop has rebound (for high *φ*). Drop splitting time on superhydrophobic surfaces mainly depends on the top width. For the hydrophobic cases, the drops after splitting remain in contact with the surface due to the high drop adhesion. On superhydrophobic surfaces, drop splitting is faster than on hydrophobic surfaces but the required critical Weber number needs to be higher. A higher drop adhesion on hydrophobic surfaces will enhance the drop friction but lower the vertical deformation, which results in easier splitting. These findings contribute to a better understanding of drop splitting in practical applications, *e.g.* icing after impact, high-efficiency tiny drop production.

## Data availability

The data supporting this article have been included as part of the Supplementary Information and Supplementary videos (ESI†). The data analysis scripts for visualizing contact angle change during drop splitting can be found at ‘https://github.com/AK-Berger/Drop-Splitting-Cas’. Extra original data and coding can be made available upon reasonable request from the readers to the corresponding authors for valid purposes.

## Conflicts of interest

There are no conflicts to declare.

## Supplementary Material

SM-021-D4SM01373E-s001

SM-021-D4SM01373E-s002

SM-021-D4SM01373E-s003

SM-021-D4SM01373E-s004

SM-021-D4SM01373E-s005
