# Supplementary material for: Drop splitting on hydrophobic wedge-shaped tips after central impact: effect of sharpness and wetting properties
Source: Soft Matter. 2025 Feb 7;21(10):1949–56. doi: 10.1039/d4sm01373e (PMC11836601; doi:10.1039/d4sm01373e)
Supplement: SM-021-D4SM01373E-s005 [file SM-021-D4SM01373E-s005.pdf]

Supplementary Information (SI) for Soft Matter.
This journal is © The Royal Society of Chemistry 2025

Supporting information

Drop Splitting on Hydrophobic Wedge-shaped Tips after Central Impact: Effect of Sharpness and Wetting Properties

Xiaoteng Zhou,[1,a),*] Diego Diaz,[1,a,b)] and Zhongyuan Ni,[1,a)] Sajjad Shumaly,[1,] Jie Liu,[3] Michael Kappl,[1,b)] Hans-Jürgen Butt[1,b)]

[1]*Max Planck Institute for Polymer Research, Ackermannweg 10, 55128 Mainz, Germany*

[2]*KTH Royal Institute of Technology, SE-100 44, Stockholm, Sweden*

[3] *Beijing National Laboratory for Molecular Science, Key Laboratory of Green Printing, Institute of Chemistry, Chinese Academy of Sciences, Beijing 100190, P. R. China.*

School of Chemical Sciences, University of Chinese Academy of Sciences, Beijing 100049, P. R. China.

a) Xiaoteng Zhou, Diego Díaz and Zhongyuan Ni contributed equally to this work.

b) Corresponding emails: kappl@mpip-mainz.mpg.de, butt@mpip-mainz.mpg.de.

*Present address: Massachusetts Institute of Technology, 77 Massachusetts Avenue, Cambridge, Massachusetts, 02139, USA

Key words: drop split, surface energy, microfluidics, mass transfer, energy harvest.

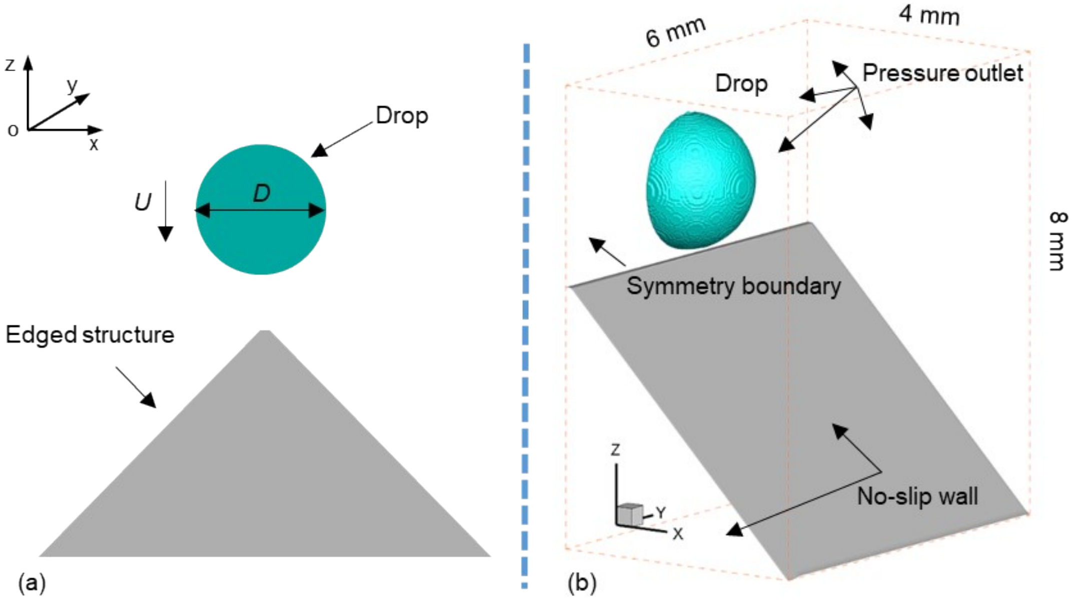

Fig. S1. (a) Schematic of drop impacting on edged structure. (b) Computational domain and boundary conditions of the simulation. At the initial time, a drop is located above the edged structure and the drop velocity is set to be a certain value.

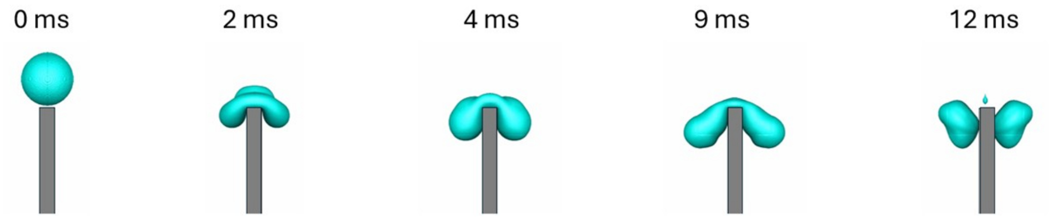

Fig. S2. VOF simulation result of drop impact and split onto an edged surface with top angle φ = 0 and top width w of 0.6 mm at We = 8 for the drop just starting to split.

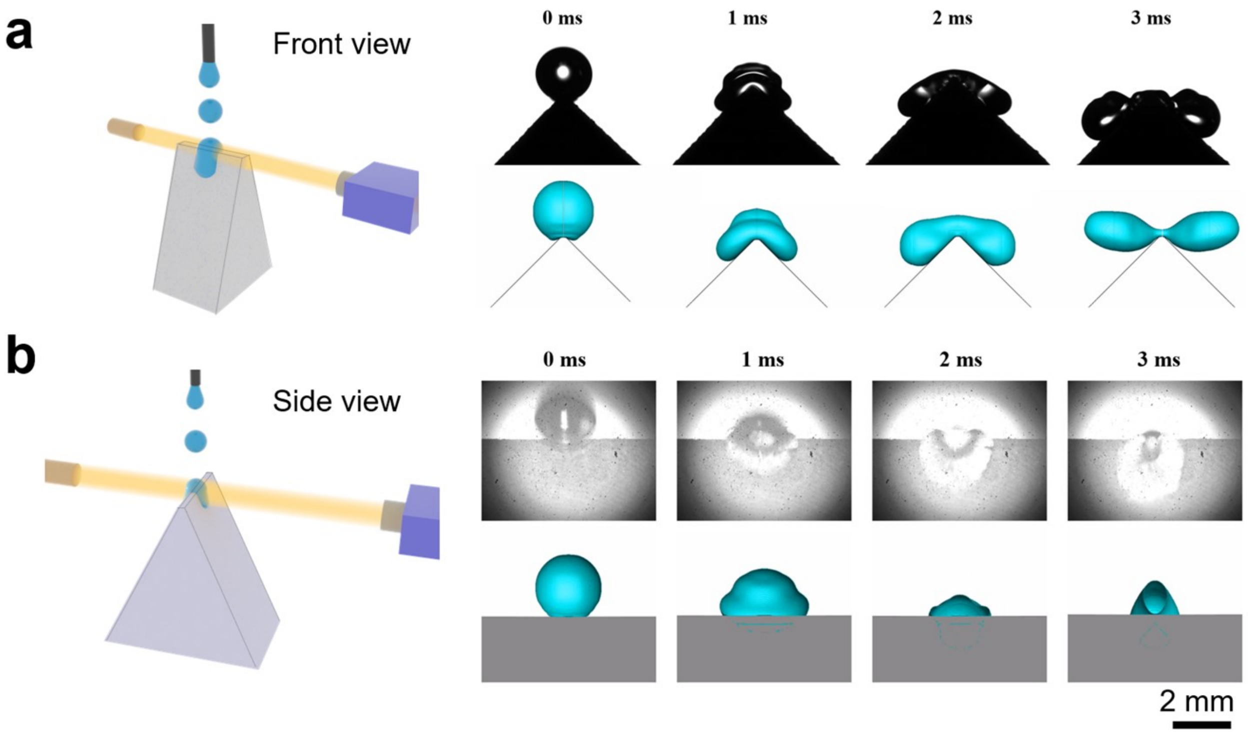

Fig. S3. Contact radius of drops changing before splitting observed from (a) front and (b) side view. The top angle φ of this case is 90°. Top width w is 0.6 mm at We = 6 for the simulation and 10 for the experiment which is close to the critical We for splitting.

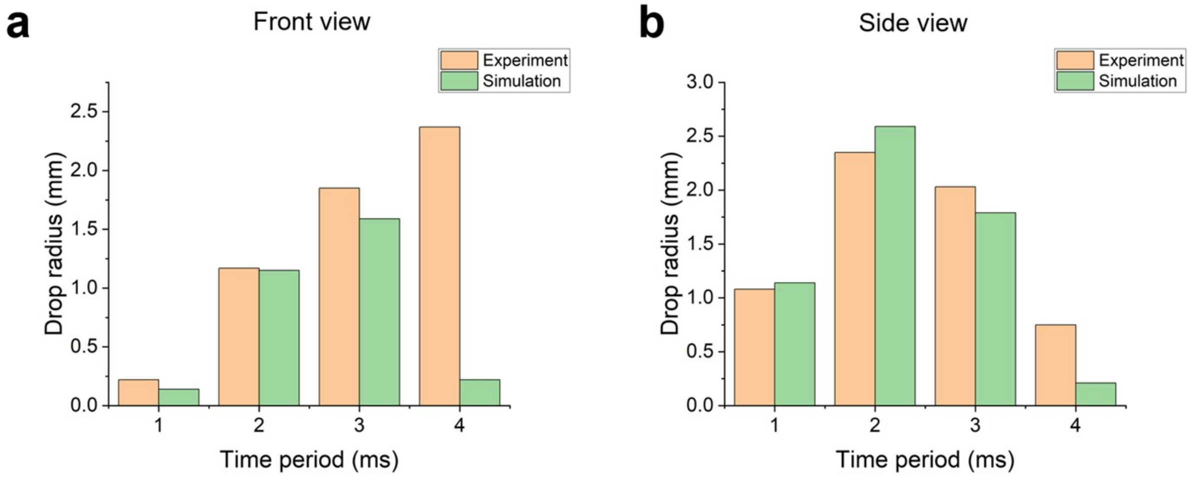

Fig. S4. The bar chart to show contact radius of drops changes before splitting measured from (a) front and (b) side view in Fig. 3.

Supplementary Section 2. Measuring the contact line dynamics during drop splitting

To measure the dynamic contact angle and its velocity as a drop splits into two parts and slides on a surface, we refined the 4S-SROF method.[1] Initially, the user must define a pixel on the baseline that marks the advancing part of the drop at the position where it first settles on the surface. This is also done for the final frame, where the two parts of the drop conclude their sliding motion on either side of the sharp shape. With this user input, the algorithm identifies the baseline and tilt angle. It then divides the video into two segments, rotating them so that the droplets appear to slide horizontally from right to left (Fig. S3). Following this adjustment, we obtained two standard videos of sliding droplets, which we can analyze using the 4S-SROF method. To reduce noise, we employed morphological transformations using the OpenCV library[2], an effective tool for preserving the shape and advancing angle of the drop while filtering out noise.

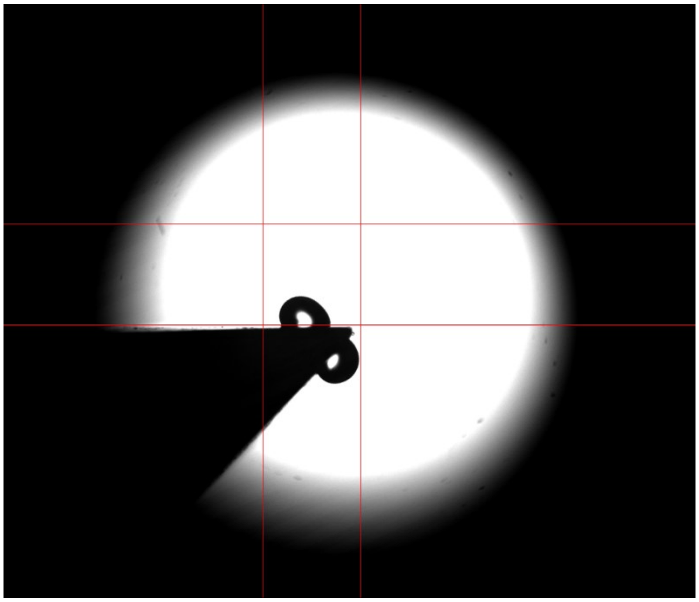

Fig. S5. One frame during the measuring of a titling angle of the whole image.

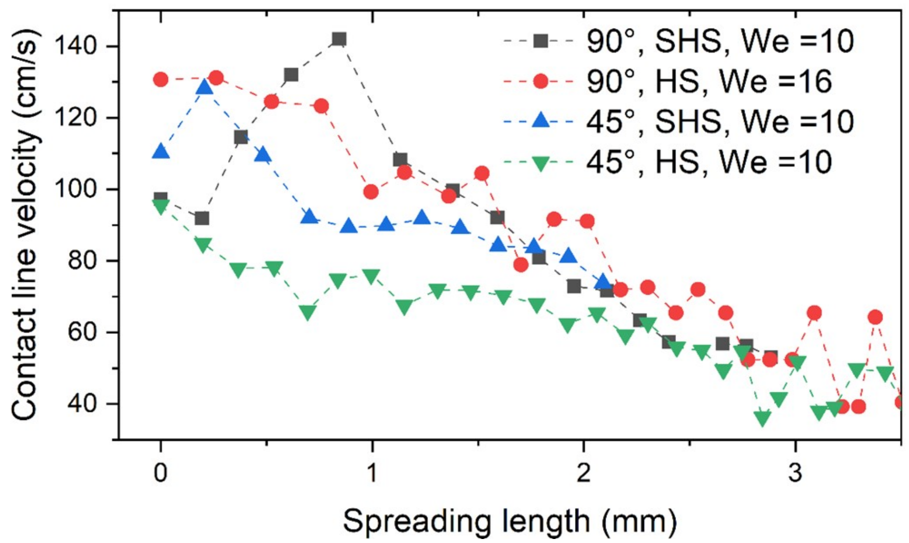

Fig. S6. Contact line velocity versus spreading length when the drop is split on superhydrophobic (SHS) and hydrophobic (HS) surfaces. The angle is the opening angle φ. The top width was 0.2 mm.

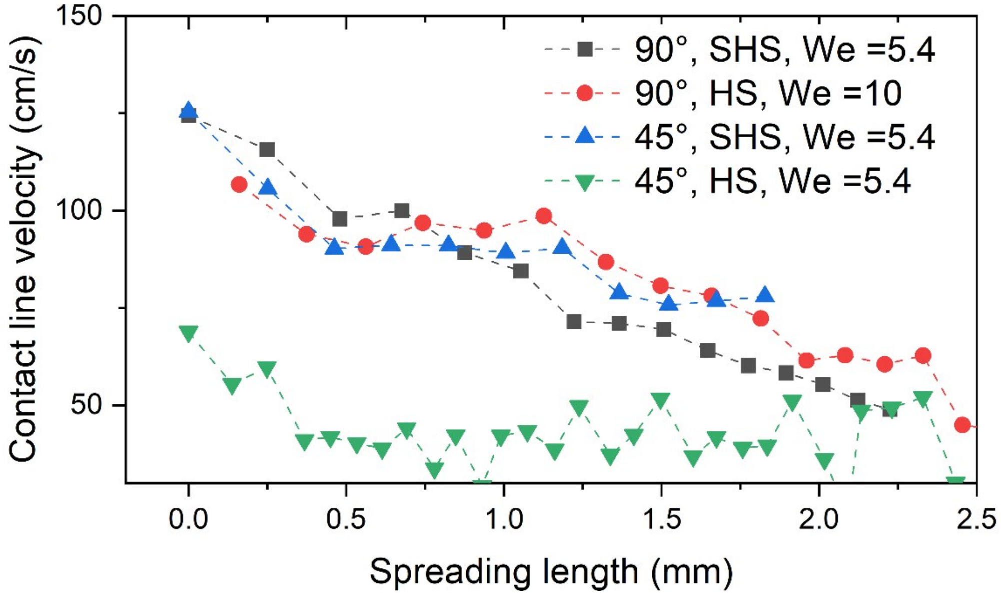

Fig. S7. Contact line velocity versus spreading length when the drop is rebounding/deposited (non-split). The top width was 0.2 mm.

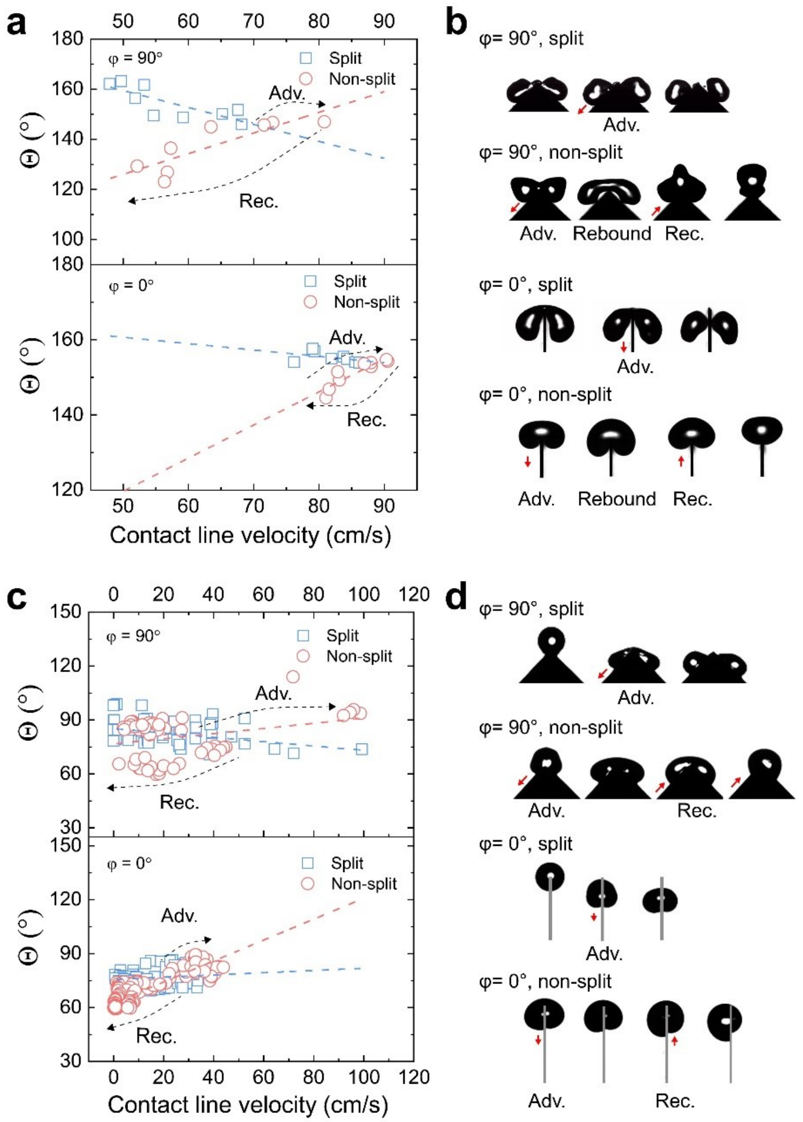

FIG. S8. Contact angle changes with the contact line velocity on superhydrophobic and hydrophobic surfaces. (a) Side-view contact angle versus contact line velocity on superhydrophobic surfaces with φ = 0° and 90°. (b) The corresponding image series of drop split or rebound process. (c) Side-view contact angle versus contact line velocity on hydrophobic surfaces with φ = 0° and 90°. (d) The corresponding image series of drop split or deposit process. Weber numbers (*We*) for these cases are set as when the drop just splits or does not split. In (a) and (c), the colored dashed line is a linear fitting of the data, and the black dashed line illustrates the advancing (adv.) or receding (rec.) process for non-split cases.

References

1. Shumaly, S.; Darvish, F.; Li, X.; Saal, A.; Hinduja, C.; Steffen, W.; Kukharenko, O.; Butt, H.-J.; Berger, R., Deep Learning to Analyze Sliding Drops. *Langmuir* **2023,** *39* (3), 1111-1122.

2. Bradski, G., The openCV library. *Dr. Dobb's Journal: Software Tools for the Professional Programmer* **2000,** *25* (11), 120-123.
